# *In vitro* Neuroprotective Potential and Lipidomics Study of Olive Leaves Extracts Enriched in Triterpenoids

**DOI:** 10.3389/fnut.2021.769218

**Published:** 2021-10-11

**Authors:** Rocío Gallego, Zully J. Suárez-Montenegro, Elena Ibáñez, Miguel Herrero, Alberto Valdés, Alejandro Cifuentes

**Affiliations:** ^1^Foodomics Laboratory, Institute of Food Science Research (CIAL, CSIC), Madrid, Spain; ^2^Departamento de Procesos Industriales, Facultad de Ingeniería Agroindustrial, Universidad de Nariño, Pasto, Colombia

**Keywords:** Alzheimer's disease, lipidomics, Aβ1–42, neuroprotection, olive leaves extracts

## Abstract

Alzheimer's Disease (AD) is the most common form of dementia that is associated with extracellular amyloid beta (Aβ) plaque formation. Genetic, environmental, and nutrition factors have been suggested as contributors to oxidative stress and neuroinflammation events that are connected to AD etiology, and secondary metabolites, such as triterpenes, have shown promising results in AD prevention. In this work, the neuroprotective and anti-inflammatory potential of an olive leaves fraction enriched in triterpenoid compounds obtained using supercritical fluid extraction (SFE) and dynamic adsorption/desorption using sea sand as adsorbent has been performed. In addition, a comprehensive lipidomics study of the response of SH-SY5Y neuroblastoma cell line to this fraction was carried out using advanced analytical methodologies, namely, charged-surface hybrid chromatography-quadrupole-time of flight mass spectrometry (CSH-Q-TOF MS/MS). The use of freely available lipidomic annotation tools and databases, and stringent cut-off filters allowed the annotation of more than 250 intracellular lipids. Advanced bioinformatics and statistical tools showed a number of phosphatidylcholines and phosphatidylethanolamines significantly increased, which could explain the protection against the cell death caused by Aβ1–42. Moreover, several triacylglycerols were found decreased. These results suggest triterpenoids from olive leaves as good neuroprotective candidates, and open a new gate for future experiments using *in vivo* models to corroborate this hypothesis.

## Introduction

Around 50 million people' worldwide' have dementia with nearly 10 million new cases every year (1). Alzheimer's Disease (AD) is the most common form of dementia and it may contribute to 60–70% of cases affecting about 24 million people. AD is characterized by progressive cognitive decline associated with a specific degeneration of cholinergic neurons and it usually begins with impairment in the ability to form recent memories, but inevitably affecting all intellectual functions. In AD, there are some hallmark pathological changes such as extracellular amyloid beta (Aβ) plaque formation and intracellular neurofibrillary tangles (NFT), that consist of an abnormally phosphorylated form of the protein Tau (τ) (2). Current evidence indicates that Aβ plaques begin to form many years before overt dementia, and genetic, environmental, and nutrition factors have been suggested as contributors of oxidative stress (3) and neuroinflammation (4) events that are connected to AD etiology. Specifically, reactive oxygen and nitrogen species (ROS and RNS), respectively, have been shown to contribute significantly to the pathogenesis and progression of AD (5–7).

Due to the physical, psychological, social, and economic impact, drug development for AD treatment is of special importance. However, only a few drugs are approved for the therapeutic treatment of AD. Most of these treatments are directed to the inhibition of cholinergic enzymes, but these drugs are also associated with unpleasant side effects (8). On the other hand, many studies have suggested that modification of lifestyle factors, such as diet, can prevent the onset of different diseases, like AD. In this sense, secondary metabolites as phenolic compounds, omega-3 fatty acids, fat-soluble vitamins, isothiocyanates, and carotenoids have shown promising results (9, 10). Moreover, other food and food by-products, such as phenolic derivatives and terpenoids from olive leaves, have been deeply investigated, showing a wide range of healthy properties such as *in vitro* and *in vivo* anti-inflammatory, antioxidant and anticarcinogenic activities (11–13). In this regard, a recently published study from our group has demonstrated that olive leaves extracts and fractions obtained using supercritical fluid extraction (SFE) and dynamic adsorption/desorption with different adsorbents have neuroprotective potential (14). This work enabled the association of *in vitro* neuroprotective bioactivities (inhibition of acetylcholinesterase (AChE), butyrylcholinesterase (BChE), and lipoxygenase (LOX) enzymes; antioxidant (ABTS) activity; and ROS and RNS scavenging capacity) to the C30 terpene content (ursolic acid derivatives, erythrodiol, and uvaol, among others). More specifically, the olive leaves fraction obtained using sea sand as adsorbent (OL-SS) showed a good blood brain barrier (BBB) permeation of its compounds compared to pharmacological AD drugs, without exerting any *in vitro* toxicity on different human cell culture lines (HK-2 and THP-1).

Lipids (and lipids metabolism) are directly related to the pathogenesis of AD because they are involved in the blood brain barrier function, the processing of the amyloid precursor protein, inflammation processes, and membrane remodeling, among other functions (15). Due to its relevance, the study of lipid changes is important to investigate alternative potential therapies against AD. In this sense, the SH-SY5Y cell line is a well stablish *in vitro* model to study neurodegenerative disorders such as AD. This cell line is of human origin, catecholaminergic, easy to maintain, and reported to be differentiable into a more mature neuron-like phenotype (16, 17). The global lipid analysis of this cell has been determined in a previous work (18), as well as the potential changes in the main mitochondrial phospholipid classes in a cellular AD-model (19). This cell line has been also used to evaluate the neuroprotective potential of different compounds, such as olive biophenols (20). However, the lipidomic study to investigate the neuroprotective effects of a natural extract enriched in terpenes has never been addressed.

The aim of the present work is to expand the knowledge on the neuroprotective and anti-inflammatory potential of the olive leaves fraction obtained using sea sand as adsorbent (OL-SS) by using different *in vitro* assays, and to study the lipidomics changes in the human neuron-like SH-SY5Y cells.

## Materials and Methods

### Chemicals and Reagents

Human THP-1 monocytes and SH-SY5Y neuroblastoma cell line were obtained from ATCC^®^ (USA); Dulbecco's Modified Eagle Medium Nutrient Mixture (DMEM/Ham's F12) culture medium, Roswell Park Memorial Institute (RPMI 1640) culture medium, fetal bovine serum (FBS), PBS, L-glutamine, antibiotic solution (including penicillin and streptomycin), antibiotic-antimycotic solution (including penicillin, streptomycin and amphotericin B), and β-mercaptoethanol were purchased from Thermo Fisher (Grand Island, NY, USA); brain-derived neurotrophic factor human (BDNF) and cholesteryl ester (CE) 22:1 were obtained from Cymit Quimica (Spain); 3-(4,5-dimethylthiazol-2-yl)-2,5-diphenyltetrazolium bromide (MTT), lipopolysaccharide (LPS) from *E. coli* O55:B5, phorbol 12-myristate 13-acetate (PMA), NaCl, β-glycerophosphate, EDTA, n-octyl-β-d-glucopyranoside (BOG), protease inhibitor cocktail, NaF, sodium metavanadate (NaVO_4_), sodium pyrophosphate, LC-MS-grade isopropanol, ammonium formate, ammonium acetate, methyl tert-butyl ether (MTBE), and toluene were obtained from Sigma-Aldrich (USA); retinoic acid (RA) was purchased from Enzo Life Sciences GmbH (Germany) and amyloid-peptide β 1–42 (Aβ1–42) was obtained from HelloBio (United Kingdom). LC-MS-grade acetonitrile (ACN) and LC-MS-grade methanol and urea were obtained from VWR Chemicals (Barcelona, Spain), whereas ultrapure water was obtained from a Millipore system (Billerica, MA, USA). Formic acid was purchased from Fisher Scientific (Waltham, MA, USA). The internal standard 12-[[(cyclohexylamino)-carbonyl]amino]-dodecanoic acid (CUDA) was purchased from LabClinics (Ann Arbor, MI, USA). The lipid standards lysophosphatidylcholine (LPC) 17:0, ceramide (Cer) d18:1/17:0, monoacylglycerol (MG) 17:0/0:0/0:0, diacylglycerol (DG) 18:1/2:0/0:0, and triacylglycerol (TG) 17:0/17:1/17:0-d5 were provided by Avanti Polar Lipids (Alabaster, AL, USA).

### Olives Leaves-Sea Sand Adsorbate

Olives leaves-sea sand adsorbate (OL-SS) tested along this work was obtained as previously described by Suárez Montenegro et al. (14). Briefly, shade-dried olive leaves were ground with a knife mill and sieved to 500–1,000 μm particle size. The adsorbent-assisted SFE process was carried out using CO_2_ at 30 MPa and 60°C for 120 min. CO_2_ was passed through the supercritical extraction cell, the extracted solute was dynamically adsorbed by the SS material, and compounds were recover after depressurization of the cell. Non-desorbed compounds remaining in the SS adsorbent were recovered by washing the SS with pure ethanol by agitation at room temperature for 2 h and passed through a 0.45 μm filter. The chemical characterization of the OL-SS by gas chromatography coupled to quadrupole-time-of-flight mass spectrometry (GC-QTOF-MS) showed uvaol (50.3%), erythrodiol (37.4%), α-tocopherol (1.5%), and β-amyrin (1.4%) as the most abundant terpenes, but squalene, α-amyrin, β-sitosterol, germacrene D, tocospiro B, and hexahydrofarnesyl acetone were also present.

### THP-1 Cell Culture Conditions and Anti-inflammatory Evaluation

Human THP-1 monocytes were cultured and maintained as described by Villalva et al. (21). Briefly, cells were grown in RPMI 1640 culture medium supplemented with 10% FBS, 100 U/mL penicillin, 100 μg/mL streptomycin, 2 mM L-glutamine and 0.05 mM β-mercaptoethanol at 37°C in 95% humidified air containing 5% CO_2_. Then, cells were seeded in 24-well-plates at a density of 5 × 10^5^ cells/mL and monocytes were differentiated to macrophages by maintaining the cells with 100 ng/mL of PMA for 48 h. After differentiation, cells were washed with PBS and incubated with 0.05 μg/mL of LPS for inducing inflammation for 24 h, with the presence of different non-toxic concentrations of the OL-SS (20 and 40 μg/mL), previously described by the group (14). Then, the supernatants were collected and frozen at −20°C. Positive controls were performed inducing cells with LPS but without sample, whereas negative controls indicated cells without LPS or extract stimulation. All conditions were performed in triplicate.

The release of pro-inflammatory cytokines including tumor necrosis factor α (TNF-α), interleukin-6 (IL-6), and IL-1β, was measured in the collected cellular supernatants using Enzyme-Linked ImmunoSorbent Assay (ELISA) kits (BD Biosciences, Aalst, Belgium), according to the manufacturer's instructions. The generated color was quantified by measuring the absorbance at 450 nm (with substrate correction at 570 nm) using a microplate reader (Bio-Tek^®^, Synergy HT, USA). The results were expressed as the mean of three determinations ± standard deviation (SD).

### SH-SY5Y Cell Culture Conditions

SH-SY5Y cells were grown, maintained and differentiated as described by de Medeiros et al. (17), with some modifications. Cells were maintained in DMEM/F12 supplemented with 10% FBS, 100 U/mL penicillin, 100 μg/mL streptomycin and 250 ng/mL antimycotic at 37°C in 95% humidified air containing 5% CO_2_. For neuronal differentiation, cells were treated with RA and BDNF. After 3–4 days of plating, cell culture medium was changed to fresh medium with 10% FBS (day 0 of differentiation). After 24 h (day 1), cell culture medium was replaced by fresh medium supplemented with 1% FBS and 10 μM of RA. After 3 days (day 4), cell culture medium was replaced by fresh medium with 1% FBS, 10 μM of RA and 50 ng/mL BDNF. Finally, 3 days after (day 7), differentiated cells were trypsinized and seeded in different well plates to perform the following experiments.

### Neurotoxicity and Neuroprotection Evaluation Using SH-SY5Y Cells

Firstly, an *in vitro* toxicity assay was performed to evaluate the OL-SS fraction on SH-SY5Y cells. Cells were plated in 24-well plates at a density of 42,000 cells/cm^2^ for 24 h. After cell attachment, different concentrations (20 and 40 μg/mL) of OL-SS were added to the cells and incubated for 24 h. The viability of the cells was then determined by the MTT assay. Briefly, the cell culture medium was carefully removed and cells were incubated with 0.5 mg/mL MTT for 3 h at 37°C. Finally, DMSO was added to solubilize formazan crystals and the absorbance was measured at 570 nm in a plate reader (Bio-Tek^®^, Synergy HT, USA). Cell viability is expressed as a percentage of living cells compared to controls (DMSO-treated). DMSO, used for diluting the OL-SS fraction, did not exceed the concentration of 0.4% (v/v). All the experiments were performed at least in triplicate.

For the Aβ1–42 neuroprotection assay, SH-SY5Y cells were plated as described above, and after 24 h, cells were pre-treated with control medium (DMEM/F12 supplemented with 1% FBS, 100 U/mL penicillin, 100 μg/mL streptomycin, 250 ng/mL antimycotic, and 0.4% of DMSO) with or without the highest non-toxic concentration (40 μg/mL) of OL-SS for 24 h. The next day, cells were incubated for another 24 h with cell medium (control), 30 μM of Aβ1–42 to evaluate the neuroprotective effect of the OL-SS fraction against this neurotoxic agent. The viability of the cells was then determined using the MTT assay as described above.

### SH-SY5Y Cell Protein Extraction and Quantification

SH-SY5Y cells were seeded in P60 plates for 24 h followed by incubation with control medium (0.4% of DMSO) with or without 40 μg/mL of OL-SS for 24 h (five plates per condition). The next day, the growth medium was removed by aspiration, cells were washed with PBS, trypsinized, and washed again with PBS. Then, cell pellets were centrifuged at 1,500 rpm for 7 min and proteins were extracted using 500 μL of the same buffer lysis buffer as in Valdés et al. (22). Samples were incubated for 60 min at 4°C, sonicated for 30 min in ice-cold water bath, and then centrifuged at 10,000g at 4°C for 15 min. The supernatants were collected, and protein concentration was quantified using Bradford assay (Bio-Rad Laboratories, Hercules, CA). Differences in protein concentration were evaluated by using a two sample *t*-test and considering significant when *p* < 0.05.

### SH-SY5Y Cell Lipid Extraction

SH-SY5Y cells were seeded and treated following the same procedure as for the protein extraction (five P60 plates per condition), and cold methanol, MTBE, and water was used to extract lipids [as in (23, 24)]. For this aim, 225 μL of methanol at −20°C containing an internal standard mixture of lysophosphatidylcholine (LPC) 17:0, ceramide (Cer) d18:1/17:0, monoacylglycerol (MG) 17:0/0:0/0:0, diacylglycerol (DG) 18:1/2:0/0:0 and triacylglycerol (TG) 17:0/17:1/17:0-d5 were added to the cell pellets. Then, 750 μL of MTBE at −20°C containing CE (22:1) were added to the samples (the concentration of each internal standard can be found in Supplementary Table 1). The samples were ground using a Mixer Mill (Retsch MM301) for 2 min at 30 s^−1^ frequency. Thereafter, 188 μL of LC-MS-grade water was added, and samples were vortexed and centrifuged at 14,000 rpm for 2 min. Finally, the upper layer (350 μL) was collected from each sample, evaporated to dryness and submitted to CSH-Q-TOF MS/MS analysis.

### Charged-Surface Hybrid Chromatography-Q-TOF Mass Spectrometry Analysis

The dried samples obtained in the previous section were resuspended in 60 μL of methanol:toluene (9:1, v/v) mixture containing 50 ng/mL of CUDA. Aliquots of 3 μL (for ESI positive) and 5 μL (for ESI negative) for five biological replicates were injected into a LC-MS/MS system consisting of a quadrupole Q-TOF series 6540 coupled to a HPLC (model 1290) both from Agilent Technologies (Germany), equipped with an Agilent Jet Stream thermal orthogonal ESI source. The Agilent Mass Hunter Qualitative Analysis software (B.10.0) was used for MS control, data acquisition, and data analysis. Compounds were separated using a Waters Acquity CSH C18 column (100 mm length × 2.1 mm id; 1.7 μm particle size) equipped with a Waters Acquity VanGuard CSH C18 pre-column (5 mm × 2.1 mm id; 1.7 μm particle size). ACN:water (60:40 v/v) was used as mobile phase (A) and isopropanol:ACN (90:10 v/v) was used as mobile phase (B) for both positive and negative modes, but 10 mM ammonium formate and 0.1% formic acid were added for positive mode, and 10 mM ammonium acetate was added for negative mode (25). The separation was performed at 65°C, and a flow rate of 0.6 mL/min was use with the same gradient as in Valdés et al. (24). The mass spectrometer was operated using the following parameters: capillary voltage of 3,500 V and with a *m/z* range from 50 to 1,700. Nebulizer pressure was set at 35 psig and the drying gas flow rate was fixed to 13 L/min and 200°C. The sheath gas flow was 12 L/min at 350°C. 175 V was chosen for the fragmentor voltage, whereas the skimmer and octapole voltage were 65 and 750 V, respectively. MS/MS analyses of three quality control pooled samples were performed employing the auto MS/MS mode using 4 precursors per cycle, dynamic exclusion after three spectra (released after 0.5 min), and collision energy of 5 V for every 100 Da. For proper mass accuracy, spectra were corrected using ions *m/z* 121.0509 (C_5_H_4_N_4_) and 922.0098 (C_18_H_18_O_6_N_3_P_3_F_24_) in ESI positive mode, and *m/z* 119.0363 (C_5_H_4_N_4_) and 980.0164 (C_18_H_18_O_6_N_3_P_3_F_24_ + acetate) in ESI negative mode, simultaneously pumped into the ionization source. Iterative MS/MS with mass error tolerance of 20 ppm and RT exclusion tolerance of ± 0.2 min was used.

### Quality Control

Quality control was assured by: randomization of the sequence; injection of three extraction method blanks and three pooled samples to equilibrate the LC-MS system before, during and after of the different sequence of samples; checking the peak shape, retention time, and intensity of the spiked internal standard; and monitoring mass accuracy of internal standards during the run.

### Data Processing

Data processing was performed as in Valdés et al. (24). Briefly, LC-MS raw data files were converted into ABF format, and MS-DIAL (v. 4.6) software (26) was used for data processing. *In-house m/z* and retention time libraries were used in addition to the public LipidBlast MS/MS spectra library (27). The following parameters were used: retention time: 0–14 min; mass range: 50–1,700 Da; MS1 tolerance: 0.01 Da; minimum peak height: 1,000 amplitude; accurate mass tolerance for mzRT library: 0.01 Da; retention time tolerance for mzRT library: 0.1 min; identification score cut off for mzRT library: 85%; accurate mass tolerance for MSP library: 0.01 Da; identification score cut off for MSP library: 80%; MS1 tolerance for alignment: 0.015 Da; retention time tolerance for alignment: 0.1 min. Peak height calculation was performed by combining data from the following detected adducts: [M+H]^+^, [M+NH4]^+^, [M+Na]^+^, [M+K]^+^, [2M+H]^+^, [2M+NH4]^+^, [2M+Na]^+^, [2M+K]^+^ adducts in positive mode; and [M–H]^−^, [M+Cl]^−^, [M+acetate-H]^−^ adducts in negative mode. The following internal standards [CUDA, LPC (17:0), Cer (d18:1/17:0), MG (17:0/0:0/0:0), DG (18:1/2:0/0:0), TG (17:0/17:1/17:0)-d5 and CE (22:1)] were used for retention time correction and for compound identification using the mzRT lipid library. The Metabolomics Standard Initiative (MSI) guidelines (28, 29) were used for metabolite annotation: MSI level 1 for metabolites with precursor *m/z, in-house* mzRT libraries and MS/MS spectral library matching; MSI level 2a for metabolites with precursor *m/z* and MS/MS spectral library matching, and MSI level 2b for metabolites with precursor *m/z* and *in-house* mzRT library matching.

### Data Post-processing and Statistical Analysis

The list of lipids was filtered by removing unknown lipids, lipids with a maximum height below three times the average height in the blank samples, and lipids with a maximum height below 1,000 units. Lipids present in three or more samples for at least one group were retained. Thereafter, missing values were imputed by half of the minimum value, and the data were processed by using MS-FLO tool (30). Duplicated lipids and isotopes were removed, and the height of the different adducts from the same compound were combined. Lipid signals were then normalized by using the sum peak height of all identified lipids (mTIC). Principal component analysis (PCA) and partial least square-discriminant analysis (PLS-DA) were performed by using MetaboAnalyst 5.0 web-based software (31), with previous “Auto scale” normalization. Exported variable importance in projection (VIP) scores were used for evaluation and PLS-DA model was evaluated according to the cross-validation of *R*^2^ and *Q*^2^. In addition, univariate analysis using the non-parametric Mann–Whitney *U*-test with FDR correction was performed, and lipids were considered significantly altered when 0.67 > FC > 1.5 and setting the FDR to 0.05.

Chemical similarity enrichment calculations were done using ChemRICH (32). Data matrices from each ionization mode were combined to generate a joint dataset. For common lipids detected in both ESI (+) and ESI (–) ionization modes, data with the highest retention time similarity (from the respective mzRT libraries), highest MS/MS similarity score, highest peak intensity, and/or better peak shape were retained. PubChem Compound Identifiers (CID) were obtained from the InChiKey or compound names by using the web-based Chemical Translation Service (http://cts.fiehnlab.ucdavis.edu/batch) (33). In addition, the simplified molecular-input line-entry system (SMILES) codes were obtained from the MSP files or from the PubChem Compound Identifier Exchange service (https://pubchem.ncbi.nlm.nih.gov/idexchange/idexchange.cgi).

## Results

### Anti-inflammatory Activity of OL-SS in Differentiated Human Macrophages

**Figure 1:**
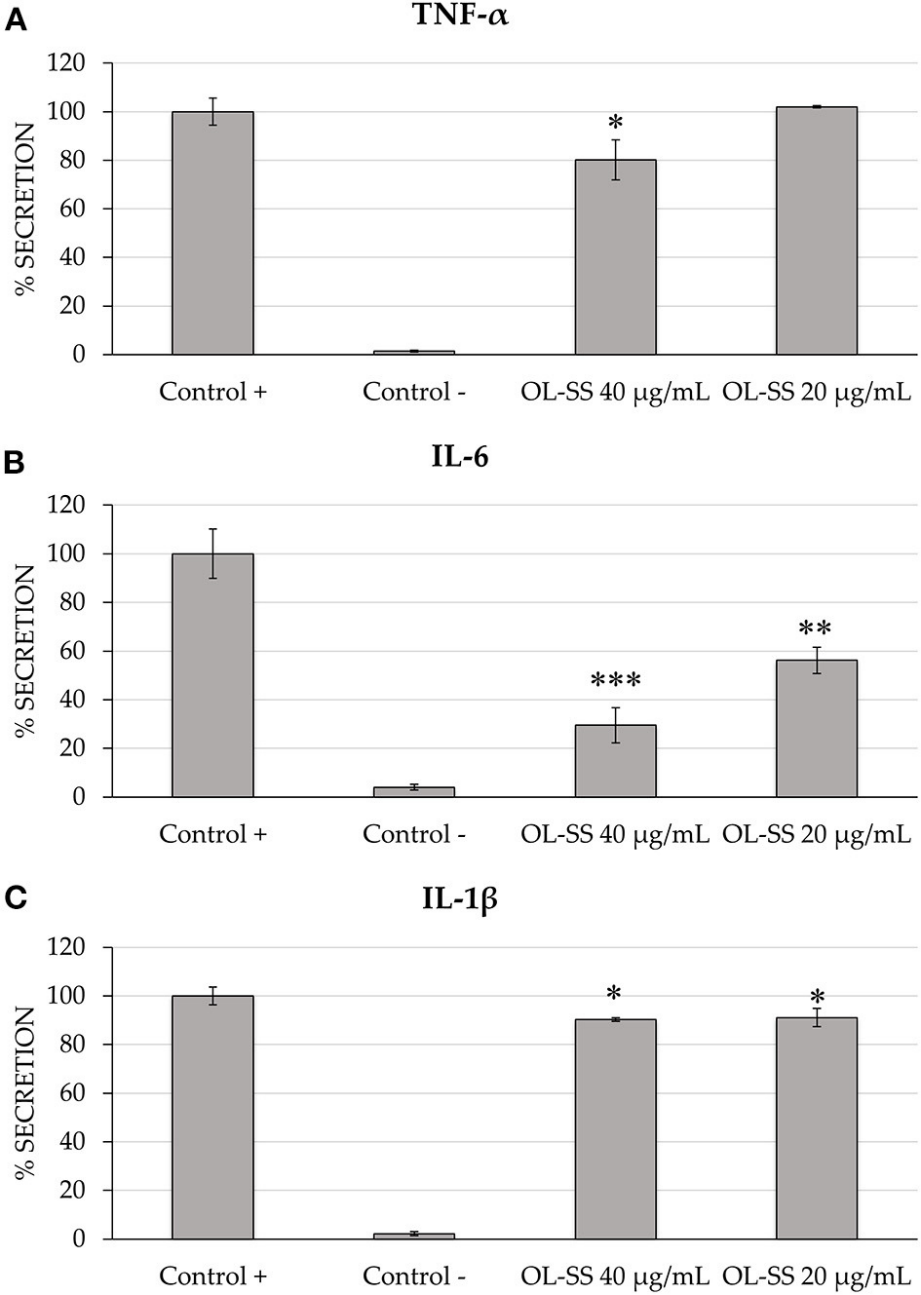
Secretion levels of TNF-α **(A)**, IL-6 **(B)**, and IL-1β **(C)** in differentiated THP-1 cells treated with and without LPS treatment (Control + and Control –, respectively), and treated with LPS in the presence of olives leaves-sea sand adsorbate (OL-SS) at 20 and 40 μg/mL. Each bar is the mean of three determinations ± SD. *Denotes statistical differences when compared with positive control (*p < 0.05; **p < 0.01; ***p < 0.001).

Differentiated THP-1 macrophages were induced to inflammation through LPS, and two non-toxic concentrations of OL-SS [20 and 40 μg/mL, as in (14)] were evaluated. As it can be observed in Figure 1, cells treated only with LPS (Control +) showed a relevant increase in all pro-inflammatory cytokines measured (TNF-α, IL-6, and IL-1β), in comparison to non LPS-induced cells (Control –) after 24 h of incubation. These LPS-induced cells were considered as the maximum cytokine secretion level (100%).

Comparing the secretion of the three cytokines, it seemed that IL-6 was the most affected one, with an important inhibition of secretion levels (43.8 and 70.5%) when 20 and 40 μg/mL of OL-SS were used, respectively, compared with levels obtained in the absence of extracts (positive control). Moreover, these two concentrations significantly reduced the secretion of IL-1β, although the decrease was lower than 10%, compared to positive control. Regarding TNF-α, only the highest concentration of OL-SS adsorbate had a significant effect, achieving a secretion inhibition of 19.2%, compared to the positive control.

### Neuroprotection Effect of OL-SS Against Aβ1–42 Using SH-SY5Y Cells

**Figure 2:**
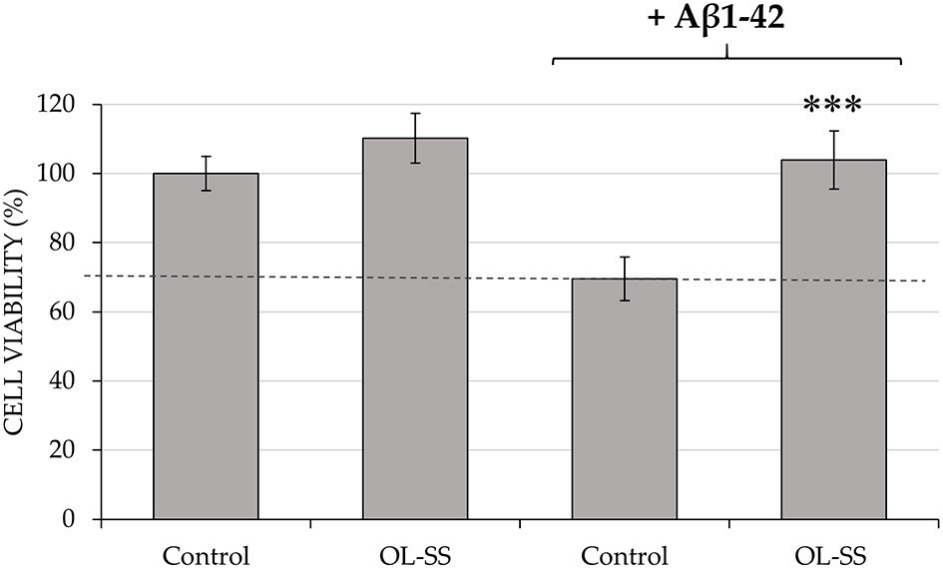
Neuroprotective effect of pre-incubation of olives leaves-sea sand adsorbate (OL-SS) against the neurotoxic agent Aβ1–42 in differentiated SH-SY5Y cells. Non Aβ1–42-treated cells were used as Control, together with only OL-SS-treated cells at 40 μg/mL. The results are mean ± SD. *Denotes statistical differences between OL-SS + Aβ1–42 and Aβ1–42 (***p < 0.001).

Firstly, an *in vitro* toxicity evaluation of the OL-SS fraction was performed on differentiated SH-SY5Y cells to future development of this extract as a natural neuroprotective agent. The same concentrations (20 and 40 μg/mL) of OL-SS used for the previous anti-inflammatory analysis were studied. Results showed that both concentrations did not significantly alter the cell viability in comparison to control cells (97.3 ± 1.7% and 110.2 ± 7.2% of cell viability for 20 and 40 μg/mL, respectively), so the highest concentration was selected to pre-treat the cells (Figure 2). Therefore, after an incubation with 40 μg/mL of OL-SS for 24 h, the neurotoxic agent Aβ1–42 was added for another 24 h at a concentration of 30 μM.

In the absence of OL-SS, the concentration used of Aβ1–42 reduced cell viability up to 30% in comparison with the control (0.4% v/v DMSO in cell medium), as it can be observed in Figure 2. However, when OL-SS was incubated with Aβ1–42, not only an increase of cell viability was achieved compared to only Aβ1–42-treated cells, but also they maintained the cell viability at maximum, reaching the same values as non Aβ1–42-treated cells.

### Protein Content in SH-SY5Y Cells

In order to carry out the comprehensive lipidomics study of the changes induced by the OL-SS treatment, five P60 cultured dishes were treated for 24 h with the 40 μg/mL of extract (diluted in 0.4% v/v DMSO) and five plates were kept in control (0.4% v/v DMSO) conditions. In parallel, the same number of cultured dishes were treated under identical conditions, and the total protein content of the two conditions was measured to complement the toxicity/viability assay results and, if necessary, for normalizing the lipid signals. In total, 35.1 ± 4.8 μg of proteins could be obtained from the control and 30.7 ± 2.7 μg could be obtained from OL-SS-treated cells, which indicates no differences in the protein content after the treatment (*p* = 0.112 after two sample *t*-test). This result confirms the absence of toxicity of the extract at the concentration studied and discards the use of the protein content to normalize the intracellular lipid signals.

### Lipidomics Study of SH-SY5Y Cells

To yield a wider view of the lipid changes during the OL-SS treatment, an untargeted lipidomics analysis on SH-SY5Y cells was performed based on CSH-Q-TOF MS/MS analysis using two different ionization modes [ESI (+) and ESI (–)], to increase the coverage of identified lipids. Data obtained from each ESI ionization mode were processed independently to avoid intensity-bias. For each of these ionization modes, the relative standard deviation of the internal standards included during sample preparation is shown in Supplementary Table 2. After data post-processing, the CSH-Q-TOF MS/MS analysis resulted in the annotation of 253 lipids: 122 in ESI (+), 63 in ESI (–) and 71 ionizing in both ionization modes (Supplementary Tables 3, 4). For those lipids annotated in both ionization modes, good Pearson correlation r values between the retention times and fold change values (*r* = 0.990 for retention time; *r* = 0.990 for fold change) were obtained (Supplementary Figure 1).

All the identified lipids were distributed in more than 15 lipid classes, and included 7 ceramides (Cer), 21 fatty acids (FA), 2 lysophosphatidylcholines (LPC), 50 phosphatidylcholines (PC), 36 ether PC, 14 phosphatidylethanolamines (PE), 31 ether PE, 3 phosphatidylglycerols (PG), 7 phosphatidylinositols (PI), 7 phosphatidylserines (PS), 17 sphingomyelins (SM), 50 triacylglycerols (TG), and 3 ether TG. As it can be observed, the most abundant classes were TG, PC, and their ether derivatives, PE and their ether derivatives, FA and SM, and the most relevant will be further discuss in the discussion section.

The PCA of data from both ESI (+) and ESI (–) ionization modes indicate that the samples are clearly differentiated between the two groups of samples (Supplementary Figure 2). Given the good separation of the PCA, a PLS-DA analysis was applied to classify the samples and to estimate the importance of each metabolite in the separation of the two groups (based on the VIP scores). After the application of the “Leave-one-out” cross-validation method, two components were selected for ESI (+) (*R*^2^ = 0.999 and *Q*^2^ = 0.980) and for ESI (–) (*R*^2^ = 0.993 and *Q*^2^ = 0.952). The *R*^2^ metric describes the percentage of variation explained by the model, and indicates that the variability of both models is well explained, and that the samples are perfectly separated (Supplementary Figure 3). On the other hand, the *Q*^2^ metric represents the predictive ability of the model, indicating that the predictive ability of both models is good. According to these models, PC (34:3, 36:4 A, 36:1, 34:0, 40:7, and 36:4 B) had the highest VIP scores which indicate that they are the most important lipids for the separation of the treated vs. control samples in ESI (+); and PG 40:7, PC 36:4 A, PC 34:3, PE O-36:5, and PC P-36:4 were the most important lipids for group separation in ESI (–) (Supplementary Tables 3, 4).

Complementary to these results, the Mann–Whitney *U*-test showed that the relative abundance of 12 and 50 lipids significantly increased and decreased, respectively, in the OL-SS treatment as compared to the control in ESI (+). Among the increased lipids, several PC (36:4 A, 34:3 B, 36:5 D, 38:6 A, 34:0, and 36:4 B) and PE (34:1, 36:2, 38:6, and 38:4) were observed, whereas 48 TG were decreased. In ESI (–), 2 lipids (PC 36:4 A and PC 34:3) and 5 lipids (PC p-36:4; or PC o-36:5, PE o-36:5, PG 36:2, FA 20:2, and PG 40:7) were significantly increased and decreased, respectively. The whole list of increased and decreased lipids is presented in Tables 1, 2, respectively.

**Table 1:** Increased lipids in SH-SY5Y cells after the treatment with olives leaves-sea sand adsorbate (OL-SS) at 40 μg/mL in comparison to control conditions after 24 h.

**Lipid name**	**Ionization mode**	**MSI level**	**Fold change**	***p*-value**	**FDR**
PC 36:4_B	ESI (+)	2b	1.541	7.91E-09	0.01187
PC 34:0	ESI (+)	1	1.546	2.35E-09	0.01187
PE 38:4	ESI (+)	2b	1.573	8.15E-09	0.01187
PI 36:2	ESI (+)	2a	1.575	1.26E-05	0.01187
PE 38:6	ESI (+)	2b	1.589	1.25E-06	0.01187
PE 36:2|PE	ESI (+)	2a	1.634	1.13E-06	0.01187
18:1_18:1					
PE 34:1	ESI (+)	2a	1.738	1.02E-07	0.01187
PC 38:6_A	ESI (+)	2b	1.822	6.09E-08	0.01187
DG 34:1	ESI (+)	2b	1.906	1.04E-05	0.01757
PC 36:5_D	ESI (+)	2b	1.989	9.89E-08	0.01187
PC 34:3_B	ESI (+)	2b	2.911	1.24E-11	0.01187
PC 36:4_A	ESI (+)	2b	4.583	3.29E-11	0.01187

*MSI, Metabolomics Standard Initiative identification; FDR, False discovery rate; DG, diacylglycerol; PC, phosphatidylcholine; PE, phosphatidylethanolamine; PI, phosphatidylinositol. Letters after lipid name indicate that one specific isomer of those contained in the database is identified*.

*Significance is determined using a Fold Change threshold ≥1.5 and a Mann–Whitney U-test with FDR <0.05*.

**Table 2:** Decreased lipids in SH-SY5Y cells after the treatment with olives leaves-sea sand adsorbate (OL-SS) at 40 μg/mL in comparison to control conditions after 24 h.

**Lipid name**	**Ionization mode**	**MSI level**	**Fold change**	***p*-value**	**FDR**
TG 52:4|TG 18:1_18:1_16:2	ESI (+)	2a	0.314	5.83E-07	0.01187
PG 40:7|PG 18:1_22:6	ESI (–)	2a	0.321	2.24E-09	0.02473
TG 58:7|TG 18:0_18:1_22:6	ESI (+)	2a	0.334	1.51E-06	0.01187
TG 54:3	ESI (+)	1	0.337	1.90E-05	0.01187
TG 52:2	ESI (+)	1	0.353	1.88E-05	0.01187
TG 60:7|TG 18:1_18:1_24:5	ESI (+)	2a	0.357	1.64E-05	0.01187
TG 56:8_B	ESI (+)	2b	0.367	9.79E-07	0.01187
TG 50:3_B	ESI (+)	2b	0.370	7.41E-08	0.01187
TG 50:1	ESI (+)	1	0.387	2.35E-05	0.01187
TG 53:3	ESI (+)	2b	0.390	2.60E-06	0.01187
TG 56:7_B	ESI (+)	1	0.391	2.42E-08	0.01187
TG 48:2	ESI (+)	1	0.394	1.13E-06	0.01187
TG 58:9	ESI (+)	2b	0.394	1.01E-05	0.01187
TG 46:1	ESI (+)	2b	0.400	3.37E-07	0.01187
TG 55:3	ESI (+)	2b	0.402	1.68E-05	0.01187
TG 50:2	ESI (+)	1	0.415	1.65E-06	0.01187
TG 58:8	ESI (+)	1	0.416	2.55E-07	0.01187
TG 48:1	ESI (+)	1	0.420	1.01E-07	0.01187
TG 52:3	ESI (+)	1	0.420	6.11E-07	0.01187
TG 51:3	ESI (+)	2b	0.424	3.37E-06	0.01187
TG 54:2	ESI (+)	2b	0.427	8.63E-06	0.01187
TG 50:3_A	ESI (+)	1	0.430	2.81E-06	0.01187
TG 58:5	ESI (+)	1	0.438	2.08E-05	0.01187
TG 56:4	ESI (+)	1	0.443	1.41E-05	0.01187
TG 56:5_B	ESI (+)	2b	0.446	2.36E-06	0.01187
TG 54:5_B	ESI (+)	2b	0.448	9.64E-07	0.01187
TG 54:4	ESI (+)	1	0.450	1.04E-06	0.01187
TG 56:3	ESI (+)	1	0.475	1.96E-05	0.01187
TG 51:2	ESI (+)	2b	0.482	1.59E-06	0.01187
TG 58:4	ESI (+)	1	0.491	1.02E-05	0.01187
TG 52:4	ESI (+)	2b	0.500	5.33E-06	0.01187
TG 48:0	ESI (+)	2b	0.510	2.37E-06	0.01757
TG O-52:2|TG O-18:1_16:0_18:1	ESI (+)	2a	0.514	2.44E-04	0.01187
TG 52:1	ESI (+)	1	0.522	6.19E-06	0.01187
TG 51:1	ESI (+)	2b	0.546	1.28E-03	0.01187
TG 60:6	ESI (+)	2b	0.553	6.85E-05	0.01187
GlcCer d42:2	ESI (+)	2b	0.554	1.10E-05	0.01187
TG 58:6	ESI (+)	1	0.559	6.77E-06	0.01187
TG 55:2	ESI (+)	2b	0.560	4.40E-05	0.01187
TG 60:3	ESI (+)	2b	0.564	6.84E-04	0.01187
TG 56:5_C	ESI (+)	2b	0.575	1.25E-06	0.01187
TG 56:6	ESI (+)	1	0.580	2.76E-06	0.01187
TG 54:1	ESI (+)	1	0.588	6.53E-06	0.01187
FA 20:2	ESI (–)	1	0.593	2.52E-03	0.02473
PG 36:2|PG 18:1_18:1	ESI (–)	2a	0.598	1.51E-05	0.02473
TG 56:2	ESI (+)	2b	0.599	1.64E-05	0.01187
TG 58:3	ESI (+)	2b	0.609	3.48E-06	0.01187
NAE 14:0	ESI (+)	2a	0.622	4.87E-06	0.01187
TG 60:5	ESI (+)	2b	0.626	5.48E-05	0.01187
TG 58:2	ESI (+)	2b	0.627	2.97E-04	0.01187
TG 46:0	ESI (+)	2b	0.630	4.36E-04	0.01187
TG 60:4	ESI (+)	2b	0.639	3.55E-06	0.01187
TG 62:7|TG 18:1_22:3_22:3	ESI (+)	2a	0.650	2.27E-04	0.01187
PE o-36:5|PE o-16:1_20:4	ESI (–)	1	0.653	2.65E-06	0.02473
PC p-36:4/PC o-36:5	ESI (–)	2b	0.665	9.25E-06	0.02473

*MSI, Metabolomics Standard Initiative identification; FDR, False discovery rate; FA, fatty acids; GlcCer, Glucosylceramide; NAE, N-Acylethanolamine; PC, phosphatidylcholine; PE, phosphatidylethanolamine; PG, phosphatidylglycerol; TG, triacylglycerol. Letters after lipid name indicate that one specific isomer of those contained in the database is identified*.

*Significance is determined using a Fold Change threshold ≤ 0.67 and a Mann–Whitney U-test with FDR < 0.05*.

**Figure 3:**
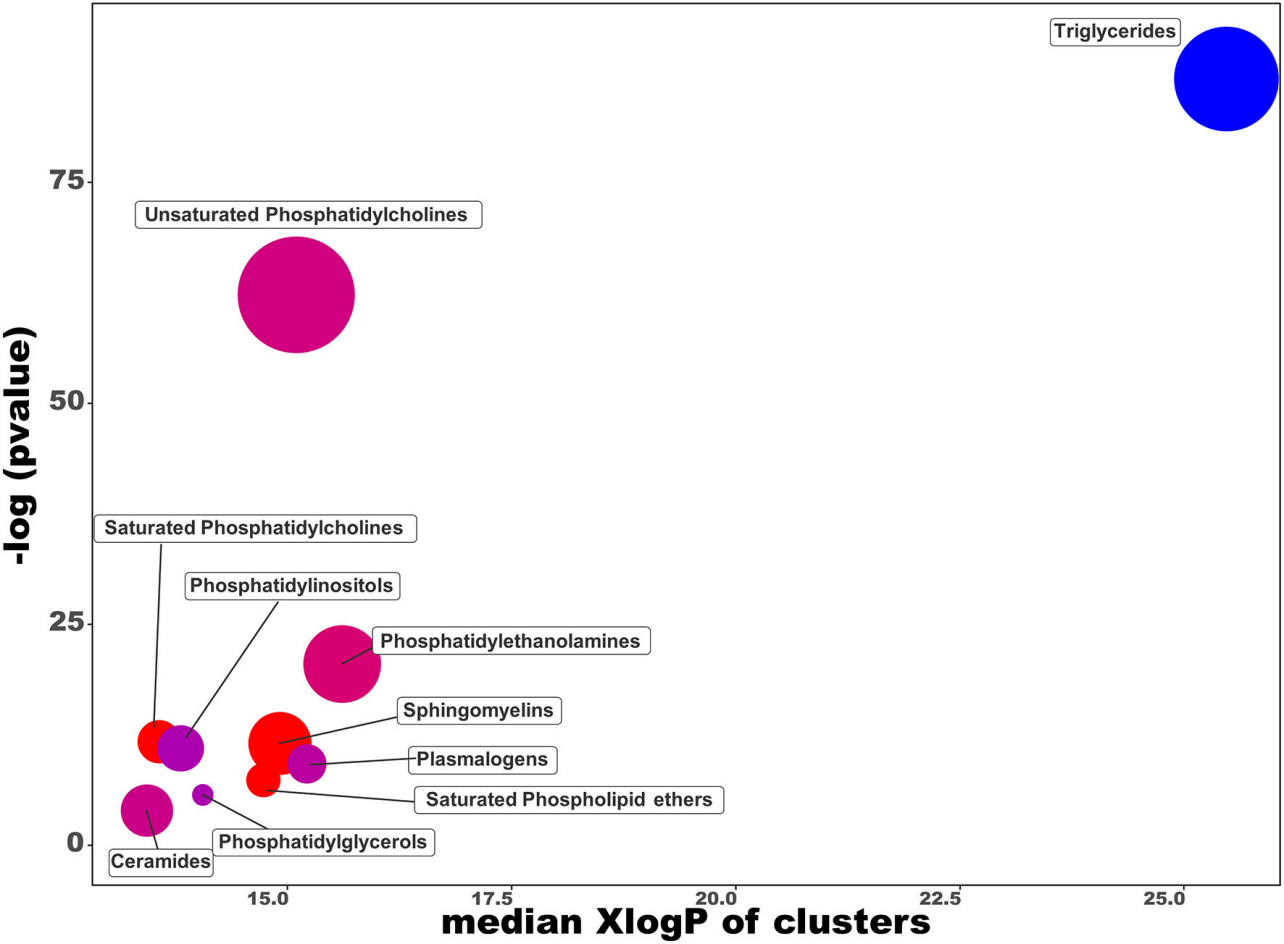
Chemical similarity enrichment analysis of lipidomics data from SH-SY5Y cells treated with olives leaves-sea sand adsorbate (OL-SS) at 40 μg/mL compared to control conditions for 24 h. The *x*-axis shows *X*log*P*-values of lipid clusters, *y*-axis shows the most significantly altered clusters on top. Clusters in red indicate an overall proportion of increased compounds, and blue clusters indicate an overall proportion of decreased compounds. Only enrichment clusters that are significantly different at *p* < 0.05 are shown.

In order to provide the chemical classes significantly altered in the intracellular lipids after the OL-SS treatment, a chemical enrichment analysis using ChemRICH was performed (Figure 3). ChemRICH is an alternative approach to the classical pathway analysis (that relies upon background databases for statistical calculations) for biological interpretation of metabolomics/lipidomics data. This statistical tool provides enrichment analysis based on structural similarity with established chemical ontologies to group metabolites/lipids, considering the fold change and statistical significance of the metabolites/lipids between the conditions studied (32). This analysis showed TG class as altered, with all the species decreased, while most of the saturated PC, SMs and saturated phospholipid ethers are increased. PI, PE, and unsaturated PC are significantly altered with some species increased, others decreased. The whole list of chemical classes and the respective *p*-values obtained is presented in Supplementary Table 5.

## Discussion

Neurodegenerative diseases such as AD are associated with numerous and complex phenomena, such as neuroinflammation, extensive oxidative/nitrosative damage caused by ROS and RNS, synaptic loss and neuronal cell death (34, 35). The release of excessive amounts of pro-inflammatory and neurotoxic mediators like ROS, IL-1β, IL-6, and TNF-α by microglia has been observed during the pathogenesis of AD, and have been related to neurodegeneration (36). Moreover, the accumulation of ROS and RNS, and the interaction between these reactive species can result in lipid peroxidation, protein oxidation, DNA damage and, ultimately, in neuronal cell death (6). In addition to these events, Aβ plaque formation and NFT are also important hallmarks of AD, and have been related with the reduction of ACh levels in the brain (37). AChE inhibitors not only increase the levels of ACh, but also reduce and prevent the formation of Aβ deposits, protecting neurons from neurodegeneration.

Although there is not an effective treatment for AD, new drugs and therapies are being developed. Among them, diet has been suggested as a key factor that can affect the development of AD, and several secondary plant metabolites have shown promising results. Previous *in vitro* studies from our laboratory have studied the neuroprotective potential of olive leaves fractions obtained from SFE coupled to dynamic adsorption/desorption with different adsorbents. In particular, the olive leaves adsorbate from sea sand (OL-SS) exhibited the highest biological activity potential in terms of neuroprotective effect, including high antioxidant capacity (based on the ABTS assay), anti-inflammatory activity (determined by LOX activity), as well as radical scavenging capacity and AChE and BChE inhibition activities (14).

One of the most critical responses to AD is due to the inflammation of microglia, so the importance of finding phytochemicals that reduce the inflammation is more than obvious. In this sense, as it was mentioned, the anti-inflammatory potential of OL-SS was previously determined by LOX enzyme inhibition assay, showing an IC50 value lower than 105 μg/mL (14). Moreover, the *in vitro* toxicity assay using THP-1 cells indicated that this fraction was not toxic up to 40 μg/mL, so that led to use of this cell line for a deeper anti-inflammatory studies.

In the present study, the secretion of three cytokines were measured after incubating the extract with the pro-inflammatory agent LPS and results showed that the highest concentration of OL-SS tested (40 μg/mL) seemed to inhibit the secretion levels from the three cytokines induced by LPS, and therefore an anti-inflammatory effect was achieved. Several studies have demonstrated the capacity of triterpenoids for modulating the immune response (38–40). For instance, the triterpenoid maslinic acid induced a significant reduction in the secretion of IL6 and TNF-α in LPS-stimulated murine peritoneal macrophages (40). Other pentacyclic triterpenes such as uvaol, erythrodiol, and oleanolic acid were demonstrated to significantly decrease IL-1β and IL-6 production in human peripheral blood mononuclear cells, whereas for TNF-α cytokine production, the three compounds reduced the cytokine secretion at high concentrations but, at low concentrations, uvaol and oleanolic acid had the opposite effect (39). Nevertheless, more specific AD-related inflammation studies could be further performed by measuring the levels of these inflammatory factors in serum, kidneys and brains of AD and LPS-induced inflammatory model mice (41) or by using transgenic models of AD, such as 5XFAD mice (42).

Among the different *in vitro* models used to study AD, the SH-SY5Y cell line has been widely used as a first step for investigating and assessing the neuroprotective effects of natural compounds because of their possible differentiation into neuron-like cells (17, 43). The *in vitro* toxicity results obtained in this study indicate that 20 and 40 μg/mL concentrations of OL-SS did not alter the cell viability of differentiated SH-SY5Y in comparison to control cells after 24 h, so the highest concentration (40 μg/mL) was selected to pre-treat the cells before the addition of 30 μM of the neurotoxic agent Aβ1–42 [the concentration used was based on previous studies (19, 44)]. As a result, the pre-treatment with OL-SS before the addition of Aβ1–42 for another 24 h was able to attenuate cell death caused by Aβ1–42. Similar results have been observed in previous studies, where the neuroprotective effect of commercial olive extracts and of individual olive biophenols against H_2_O_2_-induced cell death and against Aβ1–42-induced toxicity in SH-SY5Y cells have been reported (20). The authors observed that the exposure of SH-SY5Y cells to Aβ1–42 resulted in a decrease of cell viability and induced morphological changes, and suggested that the severe increase in ROS might be responsible. Other studies have demonstrated that terpenoids isolated from the evergreen shrub *Viburnum odoratissimum* have neuroprotective effects against H_2_O_2_-induced damage in SH-SY5Y cells through apoptosis inhibition (45); and triterpenoids isolated from the fruit peels of *Camellia japonica* were tested against Aβ-induced neurotoxicity and neuroinflammation in the mouse hippocampal HT-22 cells (46). The results of this last work indicated that these triterpenoids have strong neuroprotective effects *via* antioxidant response element gene activation and decreased the level of glutamate uptake. Other norsesquiterpenoids and triterpenoids isolated from *Populus euphratica* resins have also demonstrated neuroprotective activities in H_2_O_2_-induced HT-22 cells, and in glutamate-induced SH-SY5Y cells (47). However, the effect of triterpenes in the lipid alteration related to AD has never been studied.

The previous GC-QTOF-MS characterization of the OL-SS fraction allowed the identification of 40 terpenes and terpenoids derivatives, classified into families according to the number of isoprene units involved in the chemical structure (monoterpenoids, sesquiterpenoids, diterpenoids, and triterpenoids) (14). Among them, the triterpenoids (β-amyrin, α-amyrin, and uvaol) were associated with the neuroprotective potential of OL-SS, and they have been reported as the main compounds in olive leaves (48) with several associated biological properties (49, 50). In addition, these compounds showed promising results due to their BBB permeability, similar to pharmacological drugs as galantamine (14). It has to be noted that the OL-SS fraction is complex and more compounds present in the extract could act synergistically with the identified triterpenoids, and previous studies have demonstrated that complex olive leave extracts have higher bioactive properties due to the presence of additive and/or synergistic effects of their phytochemicals (51, 52).

Moreover, and to expand the knowledge on the possible mechanism of action of the neuroprotective effect of these compounds, a comprehensive lipidomics study was performed in this work to investigate the lipidomic response of SH-SY5Y cells to the treatment with the OL-SS fraction. The recent advances in lipid analytical methodologies, the use of different ionization modes, and the application of advanced bioinformatics tools and stringent cut-off filters have allowed the annotation of more than 250 lipids in the cell extracts and to gain an in-depth understanding on lipids.

Lipids are basic components of cell membranes and they play an important role in human health and in brain function. The brain is rich in lipids (50% of dry brain weight), and it comprises 50% phospholipids, 40% glycolipids, and 10% cholesterol, cholesterol ester, and traces of triglycerides (53). The presence of all of them is crucial for signal transduction and anchoring of membrane proteins, but their total concentrations decrease after the age of 50. The disruption of lipid homeostasis has been related to neurologic disorders as well as neurodegenerative diseases such as AD (53). As mentioned above, Aβ1–42 peptide has a key role in the pathological process of AD, and it has been shown that the APP is sensitive to cholesterol and other lipids (54, 55). In addition, different studies have shown that fatty acids and sphingomyelin modulates amyloid precursor protein (APP) metabolism and Aβ synthesis (56, 57), but the role of phospholipids in AD is not completely understood.

Our results indicate that the main lipids whose abundance was increased after the treatment with OL-SS were PCs (36:4 A, 34:3 B, 36:5 D, 38:6 A, 34:0, and 36:4 B). PCs are an essential component of cell membranes and constitute ~95% of the total choline in most tissues (58). PCs have structural roles defined primarily by chain length, and they are hydrolyzed to phosphatidate or to glycerophosphocholine and free fatty acids by phospholipase A2 (PLA2) enzymes, which have been directly associated with AD (59). PLA2s is also involved in the fluidity of cellular membranes, and because APP is a transmembrane protein, it could directly affect platelet formation (60). In this regard, previous studies have shown that PC were significantly decreased in the brain of AD post-mortem patients (61), and Gaudin et al. (62) found that PC (32:0) and PC (34:1) were decreased in senile plaques micro-extracted from the post-mortem AD brain. The authors concluded that PC regulation was affected in AD, and that it could be linked to the roles of PLA2 and phospholipase D1 (PLD1) in Aβ activation. Moreover, a recent study has demonstrated that the levels of several PC (16:0/14:0, 16:0/16:0, 16:0/18:3, 16:0/22:4, 16:0e/22:4, 18:0/18:1, 18:0/20:2, 18:1/18:1, and 18:1/20:3) were decreased in cerebrospinal fluid (CSF) from AD patients, suggesting a decline in neuronal and synaptic activity based on the increased PLA2 activity (63). In addition, an untargeted lipidomics study carried out in APP/PS1 transgenic mice have shown the decrease of PCs (p-16:0/18:3, 14:0/16:0, and 18:0/24:1) in plasma and brain of mice at the ages of 2, 3, and 7 months (64); and the levels of PCs (16:0/20:5, 16:0/22:6, and 18:0/22:6) (65) and PCs (36:6, 38:0, 38:6, 40:1, 40:2, and 40:6) (66) were found decreased in plasma from AD patients. Furthermore, Ko et al. (67) studied the neuroprotective effect of different lipids, specifically PC and PS, on cultured neurons that were later exposed to Aβ1–42. Interestingly, they found that the pre-treatment with PC had a protective effect on neurons, whereas PS or docosahexaenoic acid ethyl-ester (DHAEE) did not prevent Aβ1–42-induced neurotoxicity (67). All these results suggest that the OL-SS fraction could inhibit the activity of PLA2 and/or PLD1 enzymes, based on the increased levels of different PC species after the treatment, which might be related to the presence of triterpenoids in the olive leaves extract. However, the exact mechanism of the therapeutic effect of these triterpenoids should be investigated in a more specific study designed to unravel if the inhibition occurs, and in that case, if it occurs at the transcriptome, proteome, or phosphoproteome level.

The other group of significantly increased lipids were PE (34:1, 36:2, 38:6, and 38:4). PEs are the second most abundant glycerophospholipid in eukaryotic cells and they have diverse cellular functions such as promoting membrane fusion and fission, protein integration into membranes, or conformational changes in protein structure, and they are also associated with oxidative phosphorylation, mitochondrial stability and as important precursor of other lipids (68). The levels of PE have been also observed as decreased in the brain of AD post-mortem patients (61, 69, 70). In addition, Byeon et al. (63) observed that several PEs (16:0/22:6, 18:0/20:4, 18:0/22:4, and 18:0/22:6) and ether PE species (18:0e/22:4, 18:0e/22:6, 18:0p/22:4, and 18:0p/22:6) were decreased in CSF from AD patients, suggesting a deficiency of peroxisomes function. Similar results were observed in plasma and brain of APP/PS1 transgenic mice, where different PEs (20:3/18:0, 22:6/22:1, 22:6/22:0, p-16:0/20:4, p-18:1/20:4, and p-18:0/18:1) were decreased (64). However, the levels of other PEs (20:4/16:0, 18:1/18:2, 18:1/20:4, 22:5/22:6, and 15:0/22:4) were increased, suggesting a profound membrane remodeling during AD (71).

The global metabolic analysis also identified SM as another group of compounds whose abundance was increased. SM is the most abundant sphingolipid in the brain and is found abundantly in myelin sheaths (72). Increased levels of SM (16:0, 16:1, and 18:1) were found in the brain tissue of AD subjects that were associated with severity of AD pathology (73), and decreased levels were observed in CSF samples (63), which might be reminiscent of the amyloid-beta itself which is elevated in the brain with AD but at the same time reduced in CSF (74).

Another group of compounds whose values were significantly decreased after the treatment with OL-SS fraction is TG. As it was previously mentioned, the content of TG in the brain is really low, but TG can cross the BBB and induce central leptin and insulin receptor resistance, which might be associated with AD (75). However, conflicting reports exist in the literature regarding TG homeostasis in AD. In plasma, no relationship between AD and total TGs has been reported (76), while others suggest that elevated TGs early in life represented a risk factor for increased amyloidosis 20 years later (77). In CSF, TG were generally increased but their changes were not significant (63). And in murine models, elevated TG levels have been suggested to precede Aβ deposition (78), and several TGs (16:1/18:2/22:6, 16:1/16:1/16:1, 16:1/16:1/18:2, 16:1/18:2/20:4, 15:0/16:0/20:3, 16:0/18:0/18:2, and 16:0/18:2/20:1) have been observed increased in plasma (64). Therefore, and considering our results, we hypothesize that the decrease of TG content in neuronal cells caused by the OL-SS treatment could protect the cells against Aβ1–42 insult, but further *in vivo* studies are needed to confirm this hypothesis.

## Conclusions

In this work, and for the first time, the potential immune modulator and neuroprotective activity against Aβ1–42 of an OL-SS fraction enriched in triterpenoid compounds has been shown *in vitro*. Moreover, a comprehensive study of the lipid profile in the human neuron-like SH-SY5Y cells induced by the OL-SS fraction allowed the identification of more than 250 intracellular lipids and, through advanced bioinformatics and statistical tools, relevant findings that might explain this neuroprotective effect could be pointed out. Specifically, a great number of phosphatidylcholines and phosphatidylethanolamines were significantly increased, whereas several triacylglycerols were decreased. These results suggest triterpenoids from olive leaves as good neuroprotective candidates, and open a new gate for future experiments using *in vivo* models to corroborate this hypothesis.

## Data Availability Statement

The original contributions presented in the study are included in the article/Supplementary Material, further inquiries can be directed to the corresponding author/s.

## Author Contributions

EI, AV, and AC designed the research. AV, RG, and ZS-M designed, performed the experiments, and wrote the original draft. MH and AC acquired funding. AV and RG analyzed the data. EI, MH, and AC reviewed and edited the article. All authors contributed to the article and approved the submitted version.

## Funding

This research was funded by the Ministry of Economy and Competitiveness (MINECO), Spain, project AGL2017-89417-R.

## Conflict of Interest

The authors declare that the research was conducted in the absence of any commercial or financial relationships that could be construed as a potential conflict of interest.

## Publisher's Note

All claims expressed in this article are solely those of the authors and do not necessarily represent those of their affiliated organizations, or those of the publisher, the editors and the reviewers. Any product that may be evaluated in this article, or claim that may be made by its manufacturer, is not guaranteed or endorsed by the publisher.
