# Supplementary material for: In vitro Neuroprotective Potential and Lipidomics Study of Olive Leaves Extracts Enriched in Triterpenoids
Source: Front Nutr. 2021 Oct 11;8:769218. doi: 10.3389/fnut.2021.769218 (PMC8542692; doi:10.3389/fnut.2021.769218)
Supplement: Supplementary file 6 [file Data_Sheet_1.DOCX]

Supplementary Material

**Supplementary Figures**

**Supplementary Figure 1**: Scatterplots of retention time (**A**) and fold change values (**B**) of commonly annotated lipids by CSH-Q-TOF MS/MS (+) and CSH-Q-TOF MS/MS (-). The correlations are presented by Pearson’s correlation coefficients (r).

 **Supplementary Figure 2**: PCA score plots of data obtained by CSH-Q-TOF MS/MS (+) (A) and CSH-Q-TOF MS/MS (-) (B) after incubation of SH-SY5Y cells with OL-SS at 40 μg/mL compared to control conditions for 24 h.

 **Supplementary Figure 3**: PLS-DA score plots of data obtained by CSH-Q-TOF MS/MS (+) (A) and CSH-Q-TOF MS/MS (-) (B) after incubation of SH-SY5Y cells with OL-SS at 40 μg/mL compared to control conditions for 24 h.

**Supplementary Tables**

**Supplementary Table 1**: Concentration of internal standards used during samples preparation.

**Supplementary Table 2**: Relative standard deviation of the internal standards included during sample preparation and obtained from CSH-Q-TOF MS/MS (+) and CSH-Q-TOF MS/MS (-).

**Supplementary Table 3**: Lists of annotated lipids in CSH-Q-TOF MS/MS (+) data from SH-SY5Y cells after incubation with OL-SS at 40 μg/mL compared to control conditions for 24 h, and including the statistical values for U test and PLS-DA.

**Supplementary Table 4**: Lists of annotated lipids in CSH-Q-TOF MS/MS (-) data from SH-SY5Y cells after incubation with OL-SS at 40 μg/mL compared to control conditions for 24 h, and including the statistical values for U test and PLS-DA.

**Supplementary Table 5**: ChemRICH results obtained after comparing SH-SY5Y cells incubated with OL-SS at 40 μg/mL to control conditions for 24 h.
